# Polymer Mediated Control and Migration Effects in Spin-Crossover-Polymer Hybrids Towards Tunable Thermal Sensing Applications

**DOI:** 10.3390/polym17233226

**Published:** 2025-12-04

**Authors:** Georgios N. Mathioudakis, Georgios Kaldiris, Solveig Felton, Grace Genevieve Morgan, George A. Voyiatzis, Zoi G. Lada

**Affiliations:** 1 Foundation for Research and Technology-Hellas (FORTH), Institute of Chemical Engineering Sciences (ICE-HT), 265 04 Patras, Greece; mathioy@iceht.forth.gr (G.N.M.);; 2 School of Mathematics and Physics, Centre for Quantum Materials and Technologies, Queen’s University Belfast, Belfast BT7 1NN, UK; 3 School of Chemistry, University College Dublin, D04 C1P1 Dublin, Ireland; 4 Department of Chemistry, University of Patras, 265 04 Patras, Greece

**Keywords:** spin crossover, polymers, thermal sensing materials, migration release

## Abstract

Tailoring the spin crossover (SCO) effect in molecular materials remains a fundamental challenge, driven by the need to control critical parameters, such as the spin transition temperature (T_1_/_2_), hysteresis width, cooperativity, and switching kinetics for applications in sensing, memory, and actuation devices. SCO behavior is highly sensitive to small changes in the structure or crystal structure of the surrounding environment. In this context, achieving predictable and reproducible control remains elusive. Embedding SCO complexes into polymer matrices offers a more versatile and processable approach, but understanding how matrix–guest interactions affect spin-state behavior is still limited. In this study, we investigate a polymer-mediated strategy to tune SCO properties by incorporating the well-characterized Fe(II) complex [Fe(1,10-phenanthroline)_2_(NCS)_2_] into three polymers with distinct structural features: polylactic acid (PLA), polystyrene (PS), and polysulfone (PSF). In terms of potential electrostatic interaction between the complex and the polymeric matrixes, the polymers offer distinct features. Either there does not seem to be any specific interaction (PLA case) or, rather, there is π-π stacking between the aromatic rings of the SCO complex, and the corresponding ones present either in the backbone or in the side chain of the polymer (PSF and PS, respectively). The latter can potentially influence spin-state energetics and dynamics. Importantly, we also reveal and quantify the migration behavior of SCO particles within different polymer matrices, an aspect that has not been previously examined in SCO–polymer systems. Using magnetic susceptibility, spectroscopic, diffraction, and migration studies, we show that the polymer environment, PLA as well, actively modulates the SCO response. PSF yields lower T_1_/_2_, slower switching kinetics, and enhanced retention of the complex, indicative of strong matrix confinement and interaction. In contrast, PLA and PS composites exhibit sharper transitions and higher migration, suggesting weaker interactions and greater mobility. In addition, the semi-crystalline nature of PLA seems to induce the extension of the hysteresis width. These results highlight both the challenge and the opportunity in SCO polymer composites to tune SCO behavior, offering a scalable route toward functional hybrid materials for thermal sensing and responsive devices.

## 1. Introduction

Spin-crossover (SCO) complexes, most notably those based on Fe(II), undergo fully reversible switching between low-spin (LS) and high-spin (HS) electronic configurations when stimulated by temperature, pressure, or light [1,2,3,4,5,6,7,8]. The accompanying changes in magnetic susceptibility, color, and structural volume make these molecules attractive building blocks for advanced sensing, memory, and actuation technologies [9,10,11,12]. However, the value of SCO chemistry for real-world devices hinges on our capacity to control five key parameters simultaneously: the transition temperature (T_1_/_2_), the width and reproducibility of thermal hysteresis, the degree of elastic cooperativity, the rate of HS⇌LS interconversion, and the mechanical/processability profile of the material.

Among the major challenges, the thermodynamic sensitivity of SCO transitions is noted in particular [13,14,15]. Even small variations in ligands, packing, or intermolecular interactions can cause large shifts in T_1_/_2_ [16,17,18,19,20,21,22,23,24,25,26,27]. Achieving strong cooperativity and wide thermal hysteresis is essential for bistable behavior. However, these traits typically require rigid, crystalline lattices [11,28], while recent efforts also focus on the understanding of kinetically controlled spin transitions in bistable SCO materials [20]. On the other hand, molecular design strategies for SCO complexes towards thermoelectric applications have been attempted but these pathways require exploration of the correlation between the structures (molecular or ionic) and the metal center connected with the corresponding thermoelectric response [29]. However, the mapping of the cooperative pathways in spin crossover complexes is not profound [30]. Both elastic and electrostatic models of cooperativity, while distinct and not mutually exclusive, emphasize the importance of developing a quantitative framework to understand how intermolecular interactions govern SCO transitions. Gaining such insight would mark a major step forward in the rational design of next-generation SCO materials. In addition, when SCO complexes are incorporated into soft materials or miniaturized into nanoparticles for practical applications, this cooperativity often weakens [31]. The result is a more gradual or incomplete spin transition. Rapid HS⇌LS switching is desirable for real-time applications, yet switching kinetics are frequently slowed in viscous or disordered surroundings.

Several complementary strategies have been explored to overcome these obstacles. Molecular engineering of ligand frameworks provides predictable tuning of ligand-field strength and steric environment, enabling precise shifts in T_1_/_2_ [21,29]. Supramolecular architectures, such as coordination polymers and metal–organic frameworks, embed SCO centers in extended lattices, fostering cooperativity and guest-responsive switching [32,33,34,35]. Nanostructuring as thin films or nanoparticles accelerates kinetics and eases device integration, though often at the expense of transition abruptness; however, one of the main challenges to growing single layers on large surface areas with high crystallinity to access 2D cooperativity, which is important for building smart devices, still remains [36]. In addition to chemical host–guest interactions, mechanical coupling between SCO fillers and a polymer matrix is now recognized as a major control parameter for the transition thermodynamics and kinetics. A particularly promising route is to embed SCO complexes in polymer matrices, like PLA, PS, PSF, PMMA, TPU, or P(VDF-TrFE), which combine mechanical compliance with scalable processing and can actively modulate SCO behavior through host–guest interactions [22,24,25,37,38]. SCO switching involves a finite molecular/structural volume change, and mechano-elastic models demonstrate that volume misfit and elastic interactions control domain formation and cooperativity in dispersed particles. Experimentally, the particle expansion during spin transition is transferred to polymer composites and can produce measurable macroscopic strain and blocking stresses; the magnitude and kinetics of these effects depend strongly on particle loading, particle–matrix elastic mismatch, and dispersion, providing a direct mechanical pathway to tune T_1_/_2_, hysteresis and switching rates in SCO–polymer hybrids [22,39,40,41].

High-impact experimental evidence underscores the viability of polymer-based approaches. PLA films carrying Fe(II) SCO complexes show preserved switching and direct relevance for smart food-packaging indicators [42,43]. Ferroelectric P(VDF-TrFE) composites display spin-state-induced strain that couples to the piezo- and pyroelectric responses of the matrix, opening avenues for multifunctional electro–thermo–mechanical actuators [44]. Flexible thermochromic sensors, which are preserved inside a polymer substrate, correlate color with temperature to camera-level accuracy, demonstrating device-grade read-outs without compromising SCO integrity [44]. Beyond SCO itself, colorimetric capsule-based indicators tuned for 2–8 °C or −70 °C have been proposed for cold-chain control of mRNA vaccines, illustrating the market need for low-cost, visually read temperature tags that guarantee potency throughout distribution [45].

Despite increasing efforts to incorporate spin crossover (SCO) complexes into polymer matrices, most prior studies have focused on physical encapsulation or enhancing mechanical stability, often overlooking the chemical nature of the host environment. Polymer selection is typically driven by availability or general compatibility, rather than molecular-level design tailored to specific interactions with the SCO complex. Consequently, the influence of the matrix on SCO behavior, transition temperature (T_1_/_2_), hysteresis, and switching kinetics remains inconsistent and poorly understood across systems. In particular, the potential of non-covalent interactions, such as hydrogen bonding, π–π stacking, or dipole alignment, between polymer functional groups and SCO ligands to modulate spin-state dynamics has been underexplored. The lack of systematic comparisons between chemically distinct polymers has also hindered the development of predictive structure–property relationships. In addition to their switching properties, the potential mobility or migration of dispersed SCO particles within polymer matrices remains largely unexplored, despite its clear relevance for the stability and functionality of hybrid materials.

Here, we address these limitations by embedding a well-characterized Fe(II) SCO complex via a straightforward solution-casting approach into three industrially relevant polymers. Polylactic acid (polar, semicrystalline) can form only H bonding interactions occurring between the electrons of the O atoms of the carboxylic functional group; any compound that bears H atoms can presumably interact with PLA, and depending on the steric hindrance, these interactions may be induced or hindered. Furthermore, PLA, as a semi-crystalline polymer, hosting the complex in the crystallite-limited amorphous phase, can also affect its SCO behavior. On the other hand, polystyrene (apolar, amorphous, π-rich in the side chain), and polysulfone (aromatic, amorphous, π-rich in the backbone) can develop π-π interactions based on their aromatic groups. Therefore, the mechanical properties of the polymer matrices used in this work play an important role in determining the degree of elastic confinement experienced by the SCO particles. PLA is a semi-crystalline polyester with moderate stiffness and a glass transition temperature, allows for limited chain mobility at room temperature; polystyrene (PS), an amorphous glassy polymer, exhibits a higher *T_g_* and is therefore mechanically more rigid in the temperature range of SCO measurements; while polysulfone (PSF), a high-performance thermoplastic, is characterized by pronounced rigidity due to its aromatic backbone [46,47,48]. This design spans a broad range of interaction motifs and mechanical properties, enabling a systematic investigation of how polymer–ligand complementarity influences key SCO parameters. We hypothesize that targeted non-covalent interactions will allow rational tuning of T_1_/_2_, hysteresis width, and switching kinetics, while matrix rigidity and permeability will govern dynamic response and SCO retention under solvent exposure. The hybrid films were comprehensively characterized using variable-temperature UV–Vis and magnetic susceptibility measurements, with additional migration studies to probe polymer–SCO compatibility. By demonstrating that SCO behavior can be modulated through polymer selection, this work advances both the fundamental understanding and translational potential of responsive hybrid materials for sensing applications, including in food packaging and pharmaceutical cold chain logistics.

## 2. Materials and Methods

### 2.1. Materials and Procedures

The spin crossover sample with the chemical formula [Fe(1,10-phenanthroline)_2_(NCS)_2_] was synthesized based on previous methods [49]. Initially, to an aqueous solution of 1.3 mL containing 0.1 g of iron(II) chloride (FeCl_2_·4H_2_O), 0.2 g of hydroxylamine hydrochloride (NH_2_OH·Cl) was added to prevent the oxidation of iron(II). Then, a stoichiometric amount of 0.1 g of potassium thiocyanate (KNCS) was dissolved in 20 mL of ethanol and added to the aqueous iron(II) solution. The resulting solution is red in color. A stoichiometric amount of 0.2 g of 1,10-phenanthroline was dissolved in 30 mL of water (TDW) and added to the reaction mixture. The solution turns dark red and is cooled in a dry ice-acetone bath for 15 min, resulting in the precipitation of a red solid product. The polymers used in this study were polystyrene (PS; Code: CAS: 9003-53-6, Sigma-Aldrich, St. Louis, MO, USA), polysulfone (PSF; Code: CAS: 25135-51-7, Sigma-Aldrich) and polylactic acid (PLA) of commercial grade (Ingeo PLA 3052D by Nature Works LLC, allegedly containing ~4% D-lactide isomer) kindly supplied by ex-ARGO S.A (Gdańsk, Poland). The SCO/polymer composites were prepared following a typical non-destructive film-casting method using CH_2_Cl_2_ (dichloromethane, Code: CAS [75-09-2], from the company Scharlau, Barcelona, Spain) as solvent. In particular, 0.3 g of the respective polymer was dissolved under magnetic stirring in 10 mL of CH_2_Cl_2_. The same procedure was followed for the preparation of the composite polymers. Specifically, 0.3 g of the respective polymer was added to 10 mL of CH_2_Cl_2_, and after dissolution, 6 mg of the SCO compound was added. Then, all the solutions (polymers and composites) were poured into a Pyrex Petri dish with a diameter of 5 cm. The subsequent films of the composite polymers had a dark red color with a thickness of 100–120 μm. The films become noticeably darker, approaching a blackish color, upon cooling to the low-spin state.

### 2.2. Experimental Techniques

The morphology of the polymeric composites before and after the migration release study was determined by scanning electron microscopy (SEM). A Zeiss ZUPRA 35 VP-FEG instrument (Zeiss, Oberkochen, Germany), operating at 5−20 keV, was used for the SEM images.

Dynamic light scattering (DLS, Z-sizer) measurements were collected using a Malvern Zetasizer Nano-ZS ZEN 3600 (Malvern Panalytical, Malvern, UK) to evaluate the corresponding hydrodynamic size distribution for the SCO particles of [Fe(1,10-phenanthroline)_2_(NCS)_2_] in CH_2_Cl_2_, resembling the film-casting conditions.

For the collection of the Raman spectra at room temperature, the T64000 Horiba Jobin Yvon micro-Raman setup was used. The 632.8 nm wavelength emitted from a HeNe laser source (Optronics Technologies S.A. (Athens, Greece), model HLA-20P, 20 mW) was used for the excitation of the samples after being focused by a 50× microscope objective (Olympus (Tokyo, Japan), NA 0.55). The collected backscattered radiation was directed to the monochromator (single configuration) after passing through an appropriate edge filter (LP02-633RU-25, laser2000, IDEX Health & Science LLC, Leicester, UK) and detected by a LN-cooled CCD detector (Symphony^®^ II, Horiba Scientific, Kyoto, Japan). The instrumental calibration was carried out via the standard 520.5 cm^−1^ Raman peak position of a Si wafer. The spectral resolution was set at 5 cm^−1^.

Attenuated total reflectance–Fourier Transform Infrared (ATR-FTIR) spectra for the SCO complex, the polymeric films, and the SCO-polymer composites at room temperature were recorded on a Bruker Alpha-II with a Diamond ATR accessory from Bruker Optics GmbH (Billerica, MA, USA).

XRD characterization of all samples was carried out using an automated diffractometer (Bruker D8 Advance), equipped with a Cu tube (λCuKα = 1.54046 Å). The operating parameters were set at 40 kV and 40 mA, while the angular scanning step was set to 0.5°/min over an angular range of 5–50° (2θ).

The analysis of magnetic data allows for the calculation of critical parameters, such as the transition temperature (T_1_/_2_) between the LS and HS states, the effect of external stimuli (pressure variation and irradiation) on the stability of the spin states, and the presence or absence of hysteresis, which indicates cooperative phenomena between molecules in the solid state. In the present study, magnetic susceptibility measurements were carried out using a SQUID magnetometer (Quantum Design MPMS-XL, San Diego, CA, USA) on the polycrystalline SCO sample and on the polymer composite materials in which the SCO compound was embedded. To ensure reproducibility, magnetic susceptibility measurements were performed on three independent pieces of each composite film originating from two different casting batches (two from the same batch and one from a second batch), and all samples exhibited consistent transition temperatures, hysteresis widths, and transition profiles. The measurements were performed with the samples placed in gelatin capsules under an applied field of 5000 Oe, operating between 5 K and 300 K at a scan rate of 2 K/min. Diamagnetic corrections were applied to correct for the contribution of the diamagnetism of the samples, estimated using Pascal’s constants [50]. The measurements were performed in triplicate with high reproducibility. The spin transition temperatures T_1_/_2_ were determined from the temperature-dependent χ_M_T data by calculating the numerical first derivative d(*χ*_M_T)/dT and identifying the temperatures corresponding to the maxima of the derivative peaks during cooling and heating. These temperatures represent the midpoints of the HS→LS and LS→HS transitions, respectively. Representative derivative curves for all samples are shown in the insets of the second figure in Section 3.2. The high-spin fraction, *γ*_HS_, was obtained from the *χ*_M_T data:

(1) $$\gamma_{HS}=\frac{\chi_{Μ}Τ(Τ)-\chi_{Μ}T_{LS}(Τ)}{\chi_{Μ}T_{HS}\left(Τ\right)-\;\chi_{Μ}T_{LS}(Τ)}$$

where $\chi_{Μ}T_{HS}\;$ and $\chi_{Μ}T_{LS}\;$ are the plateaus (or average values) in the pure HS and LS states.

The temperature-domain kinetic constant $k_{T}$ was taken as the maximum absolute slope (units K^−1^). For comparison with time-domain rates, we converted $k_{T}$ to a time constant using the experimental temperature sweep rate *β* = 2 Κ/min:

(2) $$k_{t}=\beta k_{T}(s^{-1})$$

These quantities represent the characteristic switching rate and the approximate time constant under the applied temperature sweep.

Reported values correspond to the average of the heating and cooling branches unless otherwise stated.

The migration study was performed by immersing a piece of each film, with specific dimensions, in 10 mL of 50% or 20% EtOH (ethanol) in a vial. A note is made of the fact that 20% and 50% EtOH solutions are considered food simulants by the European Commission (Consolidated text of the Commission Regulation (EU) No 10/2011 of 14 January 2011 on plastic materials and articles intended to come into contact with food, amended on 16 March 2025: https://eur-lex.europa.eu/legal-content/EN/TXT/?uri=CELEX%3A02011R0010-20250316). Subsequently, the vials were placed in a thermostatically controlled incubator at 40 °C. At regular time intervals, a portion of the solution was withdrawn from the vials in order to perform the necessary UV/Vis measurements to detect substances that may have migrated from the sample. After each measurement, the removed solution was returned to the vial. Sampling and measurements were carried out over a period ranging from 1 to 30 days, depending on the sample under study. Additionally, after the above release study was completed, the films were re-immersed in the same volume of fresh solvent, and a second set of measurements was conducted on the composite polymer films over a period ranging from 4 to 14 days to investigate the total release. The detection of substances that migrated into the simulants was performed using UV–Vis spectroscopy (Shimadzu UV-1900, Kyoto, Japan) in the range of 200–800 nm, using a quartz cuvette.

## 3. Results and Discussion

### 3.1. Characterization of Precursors and Polymeric Composites

The primary conceptualization of the study relies on the dual role of the polymeric matrix to act both as the driving force toward applicability of spin crossover (SCO) complexes and, more importantly, as a critical tool for enabling selective control over SCO behavior. This includes modulation of the transition temperature (T_1_/_2_) and the nature of the transition (abrupt or gradual), which directly influences the potential of the material to function as a temperature sensor.

Accordingly, this work explores the use of three distinct polymeric matrices, each capable of establishing different types or strengths of interaction with the SCO complex. These interactions can potentially modulate or tailor the thermal spin transition behavior. Specifically, the matrices studied were polylactic acid (PLA), polystyrene (PS), and polysulfone (PSF), each selected for its characteristic interaction potential with the mononuclear iron(II) [Fe(1,10-phenanthroline)_2_(NCS)_2_] SCO complex (schematically represented in Figure S1). PLA delivers an “innocent” environment in terms of interactions, while PSF and PS are expected to interact primarily via π–π interactions with the aromatic rings of the organic ligand of the SCO complex, through their respective aromatic groups located in the main or side chains.

The SCO complex employed in this study is the well-characterized mononuclear iron(II) complex [Fe(1,10-phenanthroline)_2_(NCS)_2_], schematically represented in Figure S1. It is among the first and most extensively studied iron(II)-based SCO systems, known to undergo a thermally induced spin transition from the high-spin (HS) to the low-spin (LS) state. This transition typically occurs around 175 K and is characterized by a sharp change in spin state, with a narrow hysteresis width of less than 0.5 K.

The thermally induced spin crossover arises from the temperature-dependent contribution of entropy in the Gibbs–Helmholtz equation. At low temperatures, the entropic term is negligible, favoring the enthalpically preferred LS state. As the temperature increases, entropy becomes the dominant factor, stabilizing the HS state. SCO is inherently a sensitive and tunable phenomenon, with its properties being strongly influenced by external stimuli, such as temperature and pressure. Therefore, as stated above, the goal of the study is to acquire an easily applicable route that would enable SCO tailoring.

#### 3.1.1. Characterization via Vibrational Spectroscopy

The spectroscopic features of the complex comply with the anticipated spectroscopic bands, also indicating the successful synthesis [51,52]. These features arise from three fundamental components: the central Fe(II) ion and its Fe–N bonds, the 1,10-phenanthroline (phen) ligands, and the thiocyanate (NCS^−^) anions. The Raman and ATR/FTIR spectra of the complexes are shown in Figure S2. In the Raman spectrum, the Fe–N vibrational modes appear in the low-frequency region (90–300 cm^−1^) and are attributed to breathing-type and bending modes of the FeN_6_ octahedral core. The thiocyanate anions exhibit strong stretching vibrations in the region of 2056–2076 cm^−1^, corresponding to the N=C=S bond, while the C–H out-of-plane bending on the aromatic rings (a_2_ symmetry) is evident in the Raman spectrum at 1450 cm^−1^. In the IR spectrum of the complex, apart from the ν_CN_ bands of the thiocyanate moieties (2053 cm^−1^), the presence of the bands at 1512 and 1495 cm^−1^, associated with strong coupling of C–C and C–N stretching vibrations, was reported. The spin-state dependence of C–H out-of-plane bending modes (b_2_ symmetry mode) appeared at 723 cm^−1^, with a satellite band at 867 cm^−1^.

Subsequently, films of the individual polymer matrices PS, PLA, and PSF, as well as their corresponding SCO-containing composites (2% *w*/*w*), were characterized by ATR/FTIR spectroscopy (Figure S3). The spectra of the pure polymers confirm their structural identity through characteristic vibrational bands. In the case of polystyrene (PS), prominent peaks in the 3025–3080 cm^−1^ range correspond to aromatic CH_2_ stretching, while bands at 750–760 cm^−1^ are assigned to out-of-plane CH bending vibrations of the styrene ring. Additional features at 2850–2950 cm^−1^ indicate aliphatic stretching, and peaks at 1450 and 1490 cm^−1^ correspond to aromatic C=C vibrations. For poly(lactic acid) (PLA), the strong peak at ~1750 cm^−1^ is attributed to C=O stretching of the ester group, while bands at 1450–1500 cm^−1^ and 1350–1380 cm^−1^ correspond to CH bending and CH_3_ vibrations, respectively. The 1000–1300 cm^−1^ region features C–O–C stretching, and peaks between 2850 and 3000 cm^−1^ are linked to aliphatic CH/CH_3_ groups. The PSF spectrum is characterized by strong bands in the 1100–1300 cm^−1^ range due to symmetric and asymmetric stretching of sulfone (S=O, S–O) groups, while aromatic C–C and C–H vibrations appear between 1450–1600 cm^−1^, and aliphatic CH stretching is observed between 2850–3000 cm^−1^. Out-of-plane aromatic C–H bending modes appear in the 600–900 cm^−1^ range. In all three systems, comparison with the corresponding SCO-containing composite films reveals that the main spectral features of the polymers remain unchanged, indicating that polymer structure is preserved, also taking into account the low loading (2 wt%) of the SCO compound. While no distinct SCO peaks are observed in the PS/2% SCO and PLA/2% SCO spectra, the PSF/2% SCO spectrum exhibits a weak band near ~2050 cm^−1^, attributed to the characteristic C≡N stretching vibration of the nitrile group of the SCO.

#### 3.1.2. X-Ray Diffraction (XRD) Analysis

**Figure 1:**
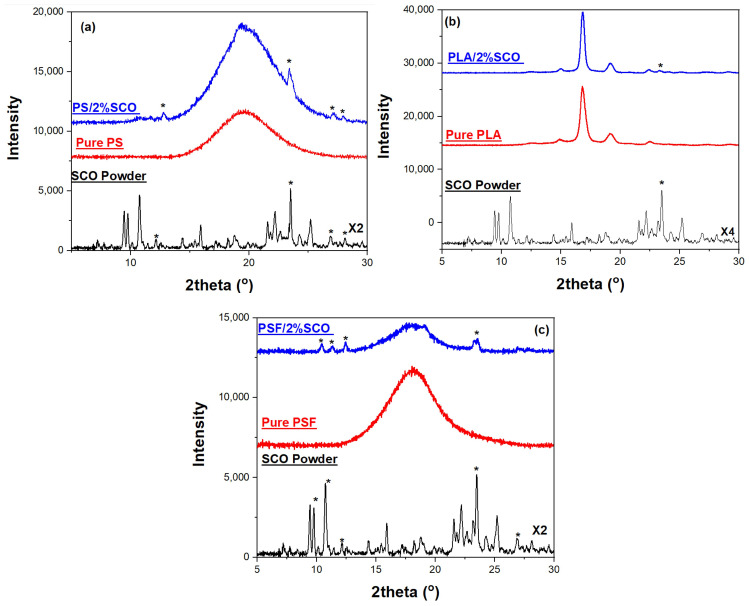
The XRD patterns of the (**a**) PS, SCO and PS/2% SCO films, (**b**) PLA, SCO and PLA/2% SCO films, and (**c**) PSF, SCO and PSF/2% SCO films; the presence of the SCO in the composite films is indicated by an asterisk.

X-ray diffraction (XRD) analysis was performed on the pure spin crossover (SCO) compound, as well as on the neat polymer films and their corresponding SCO-based composites, to assess their crystallinity and the structural integrity of the SCO phase within the polymer matrices (Figure 1). The XRD pattern of the SCO complex, [Fe(1,10-phenanthroline)_2_(NCS)_2_], exhibited numerous sharp and intense peaks in the 2θ range of 5–30°, indicative of its highly crystalline nature. In contrast, the neat polystyrene (PS) film showed a broad halo centered around 2θ ≈ 19°, characteristic of its amorphous structure, while the composite PS/2% SCO film retained this broad feature but also displayed additional weak diffraction peaks at 2θ ≈ 8.8°, 17.6°, 22.6°, 25.1°, and 27.3°. These additional reflections matched those of the pure SCO powder, particularly the peak at 22.6°, assigned to the (221) crystallographic plane, thereby confirming the incorporation and structural preservation of the SCO phase within the PS matrix despite the low loading. Similarly, neat poly(lactic acid) (PLA) exhibited a peak at 2θ ≈ 16.7°, attributed to the (200)/(110) planes of the a-form of its semi-crystalline form [53].

In the PLA/2% SCO composite, this peak was maintained alongside the emergence of a weak SCO-associated peak at 22.6°, further supporting the inclusion of crystalline SCO domains. For polysulfone (PSF), the XRD pattern displayed a broad amorphous peak at 2θ ≈ 18.6°, with the corresponding composite PSF/2% SCO again showing weak but distinguishable SCO peaks. This is also the case for PS, where the amorphous phase overlaps any contribution of SCO in the XRD of the composite. Interestingly, in all composite films, the SCO-related reflections were weaker and sometimes not the most intense, in contrast to the pure SCO powder. This attenuation likely results from a reduction in crystallite size and increased lattice strain introduced during film fabrication, which tends to broaden and weaken diffraction peaks, particularly the most intense ones. Additionally, preferential orientation of certain crystallographic planes and polymer–SCO interactions during composite formation may further modify the relative peak intensities or induce slight structural distortions or shifts.

### 3.2. The SCO Behavior in the Polymeric Composites

**Figure 2:**
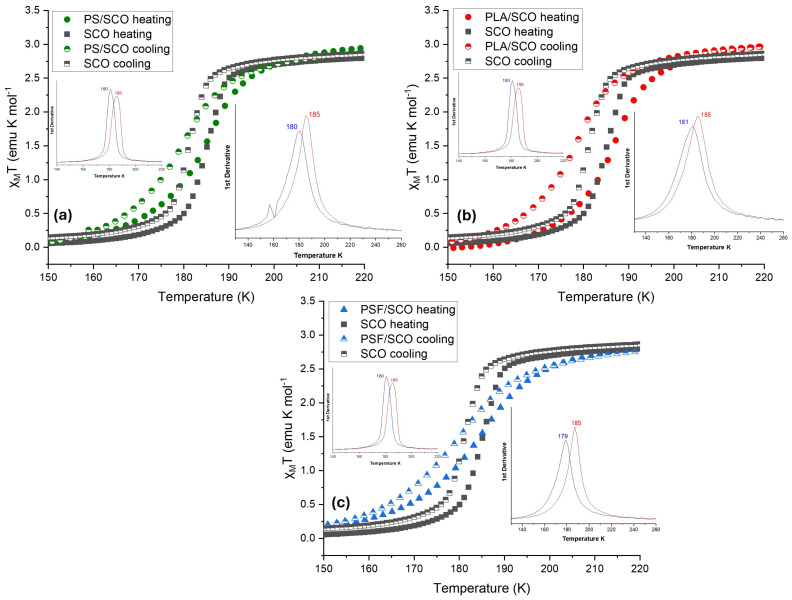
The magnetic susceptibility measurements as a function of temperature (*χ*_M_T vs. T) during heating and cooling for the (**a**) PS-SCO, (**b**) PLA-SCO, and (**c**) PSF-SCO composite (the curve of the pristine SCO material is also given for comparison); the first derivative, from which the T_1_/_2_ was calculated, is shown on the insets of each Figure (in the left inset for the SCO complex and in the right for each polymer). The broadened derivative peaks in the composites indicate a dispersion of local transition temperatures arising from microenvironmental heterogeneity within the polymer matrices.

Thermomagnetic characterization of the polymer/SCO composite films revealed significant alterations in both the thermodynamics and kinetics of the spin crossover (SCO) transition compared to the pure SCO material. As illustrated in Figure 2, temperature-dependent magnetic susceptibility (χ_M_T) measurements demonstrate the characteristic transition from the high-spin (HS) to the low-spin (LS) state upon cooling, and vice versa upon heating. For the neat SCO compound (black curves), the spin transition is relatively sharp, with half-transition temperatures (T_1_/_2_) of 180 K on cooling and 186 K on heating. Upon incorporation of the SCO material into polymer matrices such as PLA, PS, and PSF, notable deviations in the transition behavior were observed.

**Figure 3:**
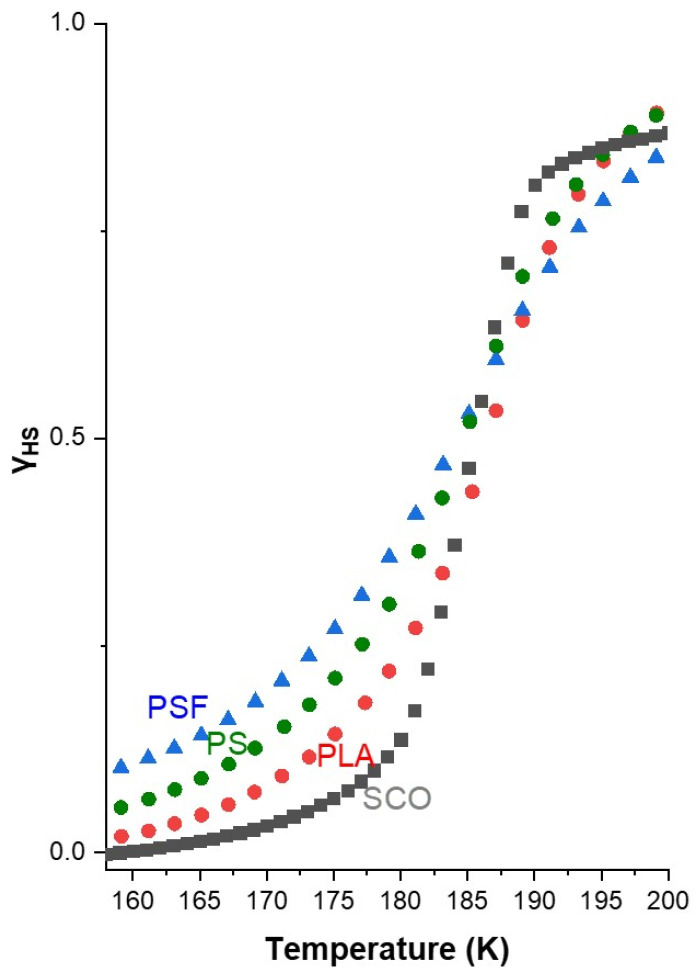
The rate of spin-state switching for the PS-SCO, PLA-SCO, and PSF-SCO films observed on the HS fraction as a function of temperature (the curve of the pristine SCO material is also provided for comparison).

In particular, the PSF/SCO composite (blue triangles) exhibited a further shift to lower transition temperatures, T_1_/_2_ = 181 K on cooling and 185 K on heating, as summarized in Table 1, indicating stronger interactions between the polymer matrix and the SCO crystals that influence the stabilization of the HS state. Additionally, the transition becomes significantly more gradual in PSF/SCO, as evidenced by the smoother spin-state switching in Figure 3. This behavior is further quantified by the rate of spin-state switching, where PSF/SCO displays the lowest kinetic constants among the studied systems (1.2500·10^−4^ s^−1^ on cooling and 1.2033·10^−4^ s^−1^ on heating), suggesting reduced cooperativity or a hindered spin transition mechanism. However, it is noteworthy that the hysteresis width is increased in the PLA/SCO film compared to the pristine SCO and the other two films. Presumably, the semi-crystalline PLA polymer can create a “matrix effect,” which can strengthen intermolecular forces in the SCO material, causing a shift in the transition temperature.

An alternative and complementary interpretation of the SCO behavior in the composites arises from the marked flattening of the χT(T) curves and the pronounced broadening of their temperature derivatives. Importantly, the thermal hysteresis is largely preserved despite this broadening, suggesting that the effect cannot be attributed solely to reduced cooperativity or slower kinetics. Instead, the data indicate the presence of a distribution of transition temperatures within each composite. This is consistent with the polymers providing a heterogeneous microenvironment, in which differences in local confinement, elastic constraints, interfacial interactions, and particle–matrix contacts generate slightly shifted T_1_/_2_ values for different subsets of SCO particles. Such environmental dispersion naturally results in a broadened overall transition, even when individual particles retain their intrinsic hysteretic behavior. This interpretation aligns well with the distinct microstructural and mechanical characteristics of PLA, PS, and PSF, each of which may impose non-uniform local stresses and free-volume conditions on the embedded SCO particles. It should also be noted that the SCO crystallite size remains essentially at the same size scale throughout processing. Dynamic light scattering (DLS) measurements performed on a CHCl_3_ dispersion of the SCO powder, under conditions resembling those used for film-casting, yielded particle sizes consistent with those observed from SEM images of both the pristine solid and the polymer-composite films (Figures S8 and S9). Therefore, the differences observed in T_1_/_2_, hysteresis width, and transition broadening among PLA, PS, and PSF may not be attributed to particle-size effects but possibly arise from matrix-dependent interaction and confinement mechanisms.

These results clearly underscore the crucial role of the polymer matrix in modulating the SCO properties. Physical and chemical characteristics of the host polymer, such as polarity, chain flexibility, and surface interaction capability, appear to directly influence the energy landscape and switching dynamics of the SCO process. The more gradual response of PSF/SCO may be advantageous in applications requiring analog tunability or enhanced operational stability during reversible magnetic switching. In contrast, the PLA/SCO and PS/SCO composites exhibit a sharper transition profile (Figure 3), suggesting their suitability for applications requiring fast and well-defined switching, similar to that of the pristine molecule. The comparative analysis shown in Figure 3 and Table 1 further confirms that the SCO transition is delayed and smoothed in the order PSF > PS > PLA, while an alteration of the hysteresis loop width is observed in the case of PLA, which will be discussed later. Thus, the choice of polymer not only affects the thermal SCO parameters but also modulates the kinetics, providing a versatile platform for tailoring functional SCO behavior.

### 3.3. Migration Release of the SCO in the Polymeric Composites

**Figure 4:**
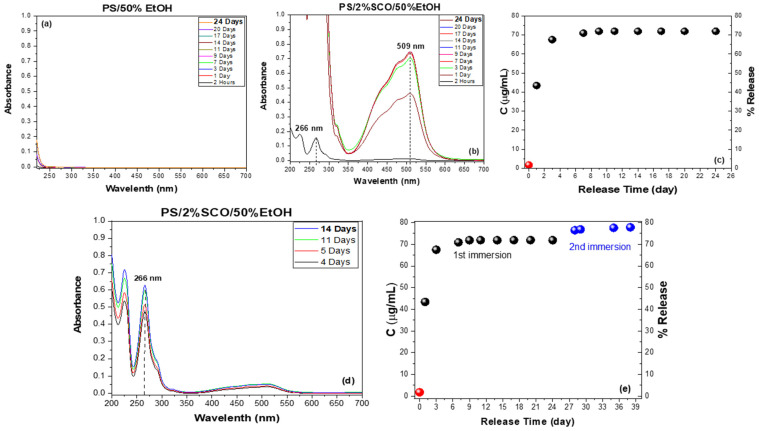
Migration release study of PS, PS/2%SCO composites in 50% *v/v* ethanol. The UV/Vis spectra at different measurement times after immersion of (**a**) PS and (**b**) PS/2%SCO films, and the corresponding concentration (μg/mL) and percentage of the SCO release in the 50% *v*/*v* ethanol as a function of time (**c**). The UV/Vis spectra at different measurement times after the re-immersion of the film (**d**), and the overall quantification of the total release deriving from both the first and second immersion (**e**). The quantification of the SCO concentration (μg/mL) for accuracy was performed using the calibration curve based either on the peak at 266 (red circle in (**c**,**e**)) or at 509 nm (black and blue circles in (**c**,**e**). The dashed lines in (**b**,**d**) act as guide for the eye.

To complement the magnetic measurements and gain deeper insight into the strength of interactions between the polymer matrix and the embedded spin crossover (SCO) complex, a migration release study was performed. This analysis aimed to assess whether the differences in thermomagnetic behavior observed among the composites correlate with the extent and rate of SCO release in ethanol-based media. Specifically, UV–Vis spectroscopic monitoring of the release from neat PS and PS/2%SCO films into 50% (*v*/*v*) ethanol was conducted under controlled conditions. The characteristic absorption bands of the SCO complex at 266 nm and 509 nm, corresponding to ligand-centered π–π* transitions and metal-centered d–d transitions, respectively, were used to track release kinetics and quantify the amount of complex released over time (Figure 4). In particular, in order to accurately record the migration percentage, when the absorption of the peak observed corresponds to less than 25 ug/mL, the stronger peak at 266 nm is taken into account, while at higher concentration release, the peak of the d–d transitions at 509 nm is considered. Calibration data and linearity at both wavelengths, obtained via standard solutions in ethanol, are provided in the Supporting Information (Figure S4). As expected, the neat PS film showed no detectable release, consistent with its chemical inertness and low solubility in the food-simulant ethanol solutions. In contrast, the PS/2%SCO composite exhibited progressive release of the SCO complex, with a rapid increase in absorbance over the first three days, followed by a plateau. Quantitative analysis indicated that approximately 72% of the initial SCO content was released during the first immersion phase. To determine whether additional material remained entrapped and the high percentage in SCO does not allow its release (poor sink conditions), the same film was re-immersed in fresh solvent, leading to a second release phase contributing an additional ~6%. The total cumulative release across both phases reached ~78%, suggesting that a portion of the SCO complex remains physically or chemically retained within the polymer network. The substantial initial release followed by limited secondary release is consistent with a moderate interaction strength between the PS matrix and the SCO complex and supports the magnetic data, where PS/SCO displayed intermediate switching kinetics.

**Figure 5:**
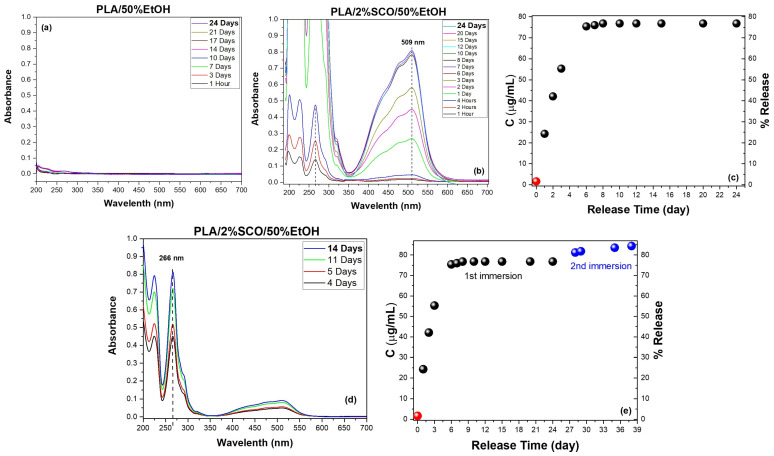
Migration release study of PLA, PLA/2%SCO composites in 50% *v/v* ethanol. The UV/Vis spectra at different measurement times after immersion of (**a**) PLA and (**b**) PLA/2%SCO films, and the corresponding concentration (μg/mL) and percentage of the SCO release in the 50% *v/v* ethanol as a function of time (**c**). The UV/Vis spectra at different measurement times after the re-immersion of the film (**d**) and the overall quantification of the total release deriving from both 1st and 2nd immersion (**e**). The quantification of the SCO concentration (μg/mL) for accuracy was performed using the calibration curve based either on the peak at 266 (red circle in (**c**,**e**)) or at 509 nm (black and blue circles in (**c**,**e**). The dashed lines in (**b**,**d**) act as guide for the eye.

PLA showed no detectable migration, confirming its stability under the release conditions. In contrast, PLA/2%SCO exhibited a progressive increase in the characteristic SCO absorbance bands, reaching ~77% release after 24 days in 50% EtOH. A second immersion yielded an additional ~7.5%, giving a total cumulative release of ~84% over 38 days (Figure 5). In our previous studies, PLA composites containing [Fe{N(CN)_2_}_2_(abpt)_2_] and [Fe(SCN)_2_(abpt)_2_] at 10% *w*/*w* (abpt = 4-amino-3,5-bis(pyridin-2-yl)-1,2,4-triazole) displayed different migration behaviors depending on the SCO complex, with total releases of approximately 40% and 90%, respectively [43], while a separate investigation with [Fe{N(CN)_2_}_2_(abpt)_2_] at 0.5% loading showed very limited release [42]. In this study, at a loading of 2% of the SCO complex, the migration release recorded was also as high as approx. 85% after 38 days. Although the results do not provide clear evidence, they could be rationalized when considering the different nature of the coordination compounds and the effect of the filler’s loading into the polymer. In the present study, the SCO complex is based on 1,10-phenanthroline, which exhibits more extended π-electron delocalization and features SCN^−^ donor groups in equatorial positions, unlike the axial coordination present in the previous systems. In parallel, the variations in loading affect the diffusion and release processes by altering the polymer packing density and available free volume.

**Figure 6:**
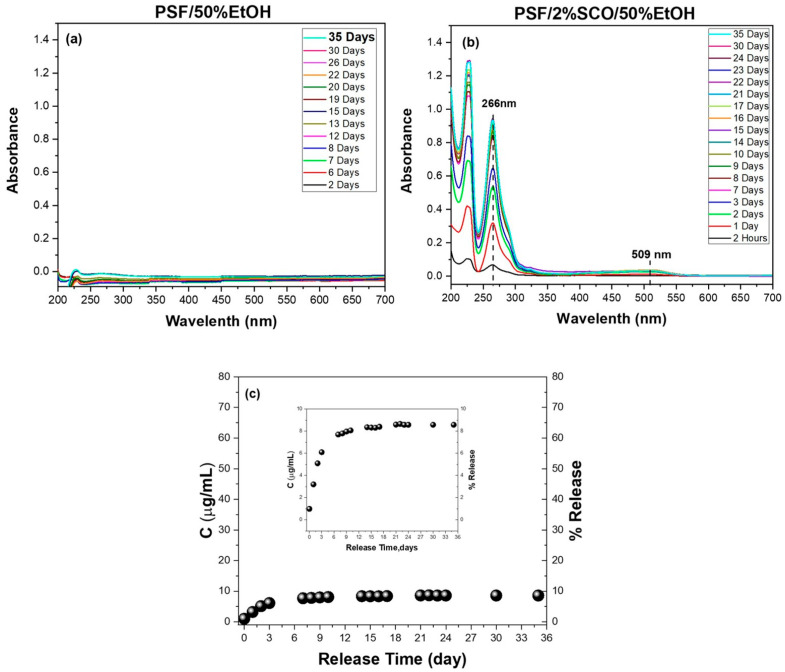
Migration release study of PSF, PSF/2%SCO composites in 50% *v*/*v* ethanol. The UV/Vis spectra at different measurement times after immersion of (**a**) PSF and (**b**) PSF/2%SCO films, and the corresponding concentration (μg/mL) and percentage of the SCO release in the 50% *v*/*v* ethanol as a function of time (**c**). The quantification of the SCO concentration (μg/mL) for accuracy was performed using the calibration curve based on the peak at 266. The dashed lines in (**b**) act as guide for the eye.

The release behavior of PSF and PSF/2%SCO films was also investigated under the same migration conditions (Figure 6). PSF showed no detectable migration, while PSF/2%SCO released only a very small fraction of the SCO complex (~9% after 35 days). This is likely a result of the denser, less permeable structure of PSF and stronger interactions between the matrix and the guest complex. The remarkably low migration rate is in agreement with the magnetic data, where PSF/SCO exhibited the most gradual and kinetically stabilized spin crossover behavior among the three polymer systems, suggesting tight confinement and reduced freedom of the SCO phase within the matrix.

These results suggest that, while PLA permits a slightly higher overall SCO migration compared to PS, the slower initial kinetics and extended-release profile may be indicative of distinct matrix–complex interactions or diffusion mechanisms specific to the semi-crystalline, biodegradable nature of PLA. Such findings align with the magnetic data, in which PLA/SCO retained relatively sharp spin crossover behavior, suggesting weaker perturbation of the SCO phase relative to PSF/SCO or PS/SCO systems. On the other hand, in the case of PLA an expansion of the hysteresis loop is observed, likely due to the influence of the semicrystalline nature of the polymer to the SCO compound, unlike the stable amorphous phase of PS and PSF.

**Figure 7:**
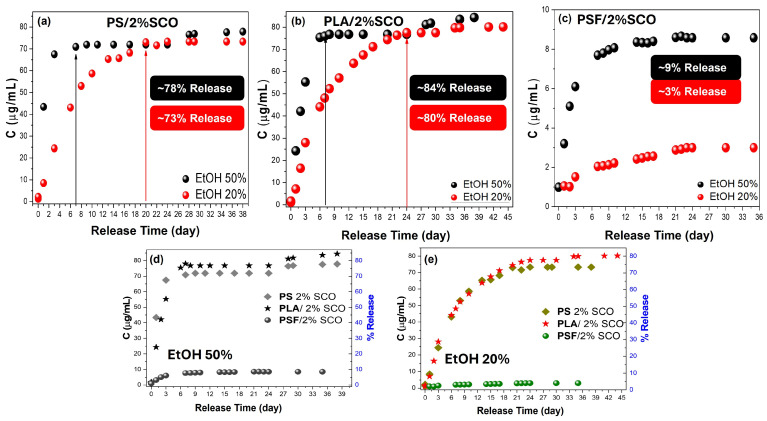
Comparison of the migration-release data between 50% and 20% ethanol for PS-SCO (**a**), PLA-SCO (**b**), and PSF-SCO (**c**) composites, and between the PS-SCO, PLA-SCO, and PSF-SCO system in 50% (**d**) and 20% ethanol (**e**).

To further elucidate the impact of solvent polarity on the release dynamics and guest–matrix interactions, a parallel migration study was carried out in 20% (*v*/*v*) ethanol (Figure S5). As expected, no detectable release occurred from the neat PS, PLA, or PSF matrices throughout the full duration of immersion in the 20% ethanol system. In contrast, the PS/2%SCO and PLA/2%SCO composites displayed progressive release of the SCO complex, with characteristic UV–Vis absorbance peaks increasing gradually over time. For PS/2%SCO, a total release of ~73% was reached after 24 days, mirroring the overall extent of release in 50% ethanol but with markedly slower kinetics and no further release upon re-immersion. Similarly, PLA/2%SCO showed a cumulative release of ~80% after 44 days, including ~2.5% released during a second immersion, again indicating a slower, more sustained diffusion profile than in 50% ethanol. These observations (Figure 7a,b,d,e) highlight the modulating role of solvent polarity, which affects diffusion rates without significantly altering the total release capacity. In sharp contrast, the PSF/2%SCO system exhibited minimal release, only ~3% after 35 days, consistent with the behavior observed in 50% ethanol (Figure 7c–e). The comparably low migration in both media for PSF-based films reflects the tighter encapsulation and stronger interactions between the SCO complex and the PSF matrix, in agreement with the suppressed SCO response and kinetic stabilization observed in the magnetic studies. Collectively, these results reinforce the influence of polymer–SCO interactions on migration behavior, with solvent polarity acting as a secondary modulator of release kinetics rather than capacity.

### 3.4. Post-Migration Structural and Morphological Analysis

To assess the structural integrity and morphological stability of the polymeric films after immersion in ethanol-based media, a comprehensive characterization was performed using ATR/FTIR (Figure S6), XRD (Figure S7), and SEM (Figure S8). The ATR/FTIR spectra of the neat and composite films before and after immersion in both 20% and 50% ethanol revealed no significant changes in the positions of the characteristic absorption bands of the polymer matrices, suggesting that neither chemical degradation nor substantial hydrolysis occurred during the course of the release studies. In the case of the PSF-SCO composite, the weak contribution of the SCO peak at 2052 cm^−1^, assigned to the symmetric stretching vibration of the N≡C–S moiety, was not discernible despite the low release efficiency observed in this system. In addition, the FTIR spectrum of the immersed PSF film shows a new peak at ~1650 cm^−1^, accompanied by a wide O–H stretching envelope between 3200 and 3500 cm^−1^. These features are characteristic of the H–O–H bending vibration of absorbed or strongly bound water, whose frequency shifts toward higher wavenumbers when water is hydrogen-bonded or confined within the polymer matrix.

X-ray diffraction patterns provided additional insights into the phase behavior of the films following exposure to ethanol. As expected, PS and PSF exhibited amorphous patterns, which remained unaltered after immersion. Notably, minor reflections attributable to the crystalline SCO complex were faintly visible in the PSF-SCO samples similarly before and after the migration study. After immersion, the neat PSF film exhibited an additional low-angle diffraction feature at around 2θ ≈ 10°, along with the characteristic amorphous halo near 20°. The halo at ~20° corresponds to the average interchain distance (d ≈ 4.4–5.0 Å) typical of the amorphous PSF matrix, whereas the new broad feature at ~10° (d ≈ 8.8 Å) indicates the formation of a longer-range periodicity attributed to the structural rearrangement upon hydration, reflecting increased spacing between polymer chains and partial ordering of polymer–water domains. While no literature source reports exactly these diffraction features in hydrated PSF, the interpretation that this feature reflects increased inter-chain spacing/partial ordering of polymer–water domains is consistent with SAXS/SANS studies of hydrated ionomeric and aromatic polymer membranes, which routinely show hydration-induced low-q (ionomer/matrix) peaks reporting domain spacings in the 1–5 nm range [54]. PLA exhibited a prominent diffraction peak at 16.7°, corresponding to the (110)/(200) planes of its α-crystalline phase, while upon immersion in 50% ethanol, this peak displayed a reduction in intensity and broadening, indicative of partial disruption of crystalline order, likely due to solvent-induced lattice strain or decreased molecular packing efficiency. These observations suggest that while ethanol did not degrade PLA chemically, it did affect its crystallinity and could influence guest release through subtle structural rearrangements. The presence of SCO in the PLA composite was not evident either before or after migration.

To investigate potential morphological changes and the distribution of the SCO compound within the polymer matrices, cross-sectional cryo-SEM was conducted before and after immersion. For both PS-SCO and PLA-SCO films, SEM images revealed the appearance of voids or cavities following SCO release. The PSF-SCO system showed no detectable changes in morphology even after extended immersion, indicating stronger physical confinement of the guest material.

Taken together, these results suggest that the release of the SCO compound occurs primarily through physical mechanisms, such as surface extraction and internal diffusion, without polymer degradation. The extent and rate of release are strongly modulated by the structural characteristics of the polymer and the distribution of the guest phase, with PSF providing the highest degree of retention and morphological preservation, while PLA and PS exhibit progressive depletion of the SCO compound in correlation with observable microstructural disruption. For PLA, the sharper albeit increased hysteresis width may prove particularly interesting, and in future work, the influence of molecular orientation after stretching will be investigated, which will most probably further influence both thermomagnetic and release properties [55].

## 4. Conclusions

This study highlights how the polymer matrix affects the properties of spin crossover (SCO) compounds, both at a fundamental and applied level. The results suggest that targeted modulation of SCO thermomagnetic behavior is feasible by tailoring the interactions between the functional groups of a polymer and the organic ligand of a spin crossover compound. It was found that the polymer matrix plays an active role in modulating both the thermodynamic stability and the kinetics of the SCO process. Deviations in T_1_/_2_ across all composites compared to the pristine SCO confirmed that even low concentrations of polymer can strongly influence magnetic behavior through specific interactions. Notably, the PSF/SCO film showed a gradual switching behavior, consistent with strong π–π interactions between the sulfone polymer and the aromatic moieties of the complex. Moreover, the migration study served not only to assess the system’s stability under application-relevant conditions but also as an independent indicator of SCO–polymer interaction strength. The migration study in ethanol media revealed distinct SCO release profiles among PS, PLA, and PSF composites. While PS and PLA composites released ~78–84% of the embedded SCO complex over time, the PSF matrix exhibited minimal release (~9%), indicating stronger confinement and interaction, consistent with its kinetically stabilized spin crossover behavior. The broadening of the hysteresis width in the case of the PLA-incorporated SCO compound needs further attention and detailed investigation. Collectively, the results establish a clear correlation between the strength of polymer–SCO interactions, magnetic switching behavior, and migration performance, demonstrating that the polymer matrix serves not only as a carrier but also as a functional component capable of tuning the performance and stability of SCO materials. This insight provides a valuable framework for the design of responsive and application-specific SCO-based composites.

## Figures and Tables

**Table 1:** The kinetic constants from the analysis of the spin-state switching rate and the corresponding spin crossover transition temperatures (T_1_/_2_) during the cooling and heating of the composite films and the SCO compound; the hysteresis width [T_1/2_ (heating) − T_1/2_ (cooling)] was also easily calculated. Values are reported as mean ± standard deviation from three independent χ_M_T(T) scans.

Sample	Kinetic Constant (×10^−3^)/K^−1^	k_t_ (×10^−4^)/s^−1^	T_1/2_/K	Hysteresis Width
SCO	3.85 (cooling) ± 0.002 3.83 (heating) ± 0.003	1.2833 (cooling) 1.2767 (heating)	T_1/2_ (cooling) = 180 K T_1/2_ (heating) = 186 K	6 K
PS/SCO	3.82 (cooling) ± 0.003 3.80 (heating) ± 0.002	1.2733 (cooling) 1.2667 (heating)	T_1/2_ (cooling) = 180 K T_1/2_ (heating) = 185 K	5 K
PLA/SCO	3.88 (cooling) ± 0.003 3.77 (heating) ± 0.002	1.2933 (cooling) 1.2567 (heating)	T_1/2_ (cooling) = 179 K T_1/2_ (heating) = 187 K	8 K
PSF/SCO	3.75 (cooling) ± 0.003 3.61 (heating) ± 0.002	1.2500 (cooling) 1.2033 (heating)	T_1/2_ (cooling) = 181 K T_1/2_ (heating) = 185 K	4 K

## Data Availability

The original contributions presented in this study are included in the article/Supplementary Materials. Further inquiries can be directed to the corresponding authors.

## Supplementary Materials

The following supporting information can be downloaded at: https://www.mdpi.com/article/10.3390/polym17233226/s1. Figure S1: The chemical structure of the coordination compound [Fe(1,10-phenanthroline)_2_(NCS)_2_]; Figure S2: The Raman (a) and ATR/FTIR of the complex [Fe(1,10-phenanthroline)_2_(NCS)_2_]; Figure S3: The ATR/FTIR spectra of the (a) PS, SCO, and PS/2% SCO films, (b) PLA, SCO, and PLA/2% SCO films, and (c) PSF, SCO, and PSF/2% SCO films; Figure S4: (a) UV/Vis spectra of the complex [Fe(1,10-phenanthroline)_2_(NCS)_2_] in 20 or 50% ethanol. The calculated maximum migration is at 100 μg/mL, which corresponds to an absorption of less than 1. (b) The calibration curve of [Fe(1,10-phenanthroline)_2_(NCS)_2_] in 20 or 50% ethanol; Figure S5: Migration release study of PS, PLA, and PSF and the PS-SCO, PLA-SCO, and PSF-SCO composites in 20% *v*/*v* ethanol. The UV/Vis spectra at different measurement times after immersion of (a) PS, (b) PS-SCO, (d) PLA, (e) PLA-SCO, (g) PSF, and (h) PSF-SCO, and the corresponding concentration (μg/mL) and percentage of the SCO release in the 20% *v*/*v* ethanol as a function of time (c, f, i); Figure S6: ATR/FTIR spectra before and after migration of PS and PS-SCO (a), PLA and PLA-SCO (b), and PSF and PSF-SCO (c) composites; Figure S7: XRD before and after migration of PS and PS-SCO (a), PLA and PLA-SCO (b), and PSF and PSF-SCO (c) composites; Figure S8: SEM images of the SCO particles of [Fe(1,10-phenanthroline)_2_(NCS)_2_], before and after migration of PS-SCO, PLA-SCO, and PSF-SCO composites; Figure S9: Calculated particle size distributions (with respect to intensity) obtained for the SCO particles of [Fe(1,10-phenanthroline)_2_(NCS)_2_] in CH_2_Cl_2_, resembling the film-casting conditions. The extracted average hydrodynamic radius of SCO particles is denoted in the corresponding plot.
