# Supplementary material for: Polymer Mediated Control and Migration Effects in Spin-Crossover-Polymer Hybrids Towards Tunable Thermal Sensing Applications
Source: Polymers (Basel). 2025 Dec 4;17(23):3226. doi: 10.3390/polym17233226 (PMC12694122; doi:10.3390/polym17233226)
Supplement: Supplementary file 1 [file polymers-17-03226-s001.zip › polymers-4010024-supplementary.pdf]

Supporting Information

S1. The structure of [Fe(1,10-phenanthroline)$_2$(NCS)$_2$]

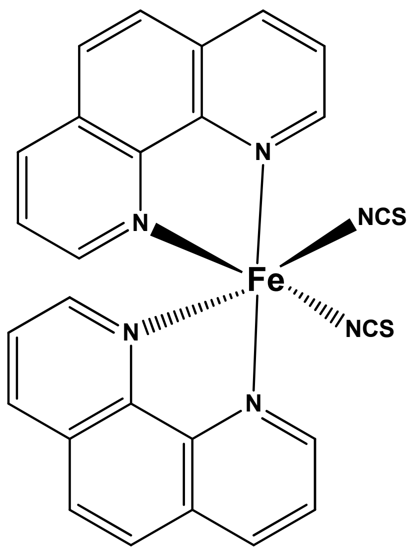

Figure S1. The chemical structure of the coordination compound [Fe(1,10-phenanthroline)$_2$(NCS)$_2$].

S2. Vibrational analysis of the SCO compound

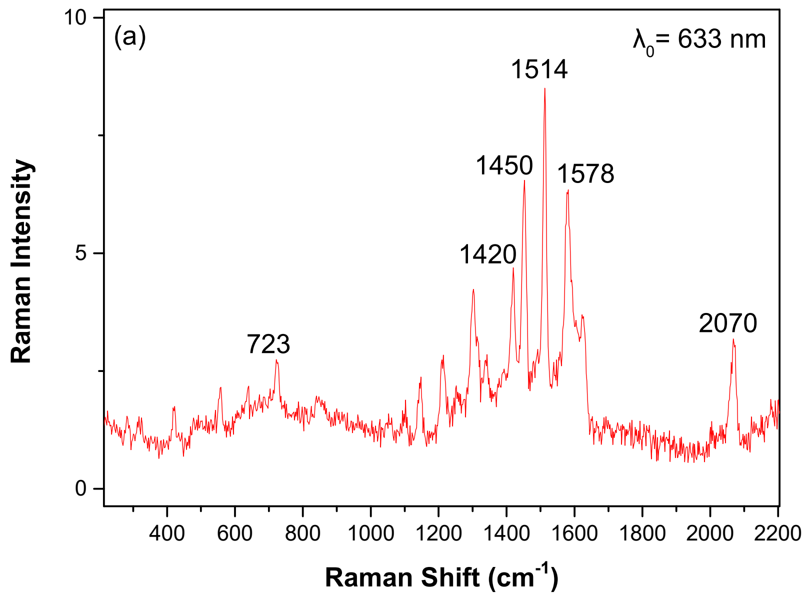

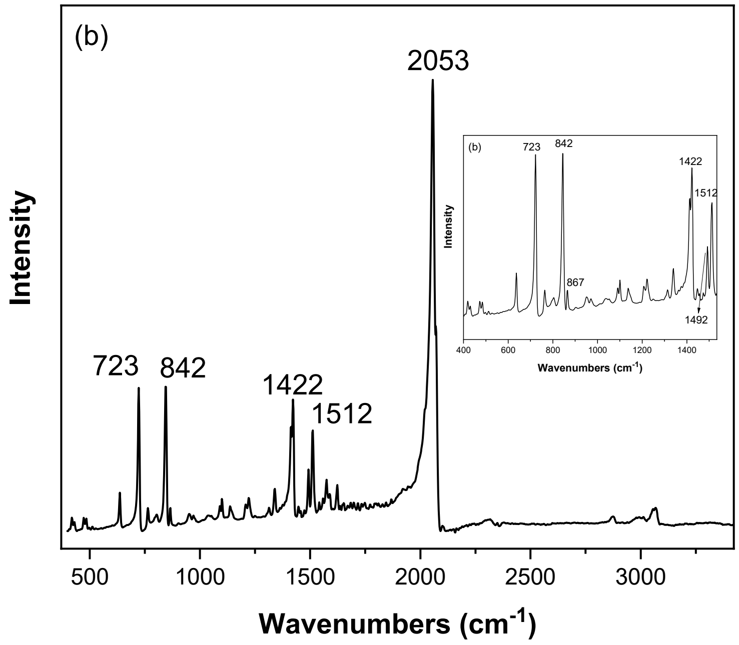

Figure S2. The Raman (a) and ATR/FTIR of the complex [Fe(1,10-phenanthroline)$_2$(NCS)$_2$].

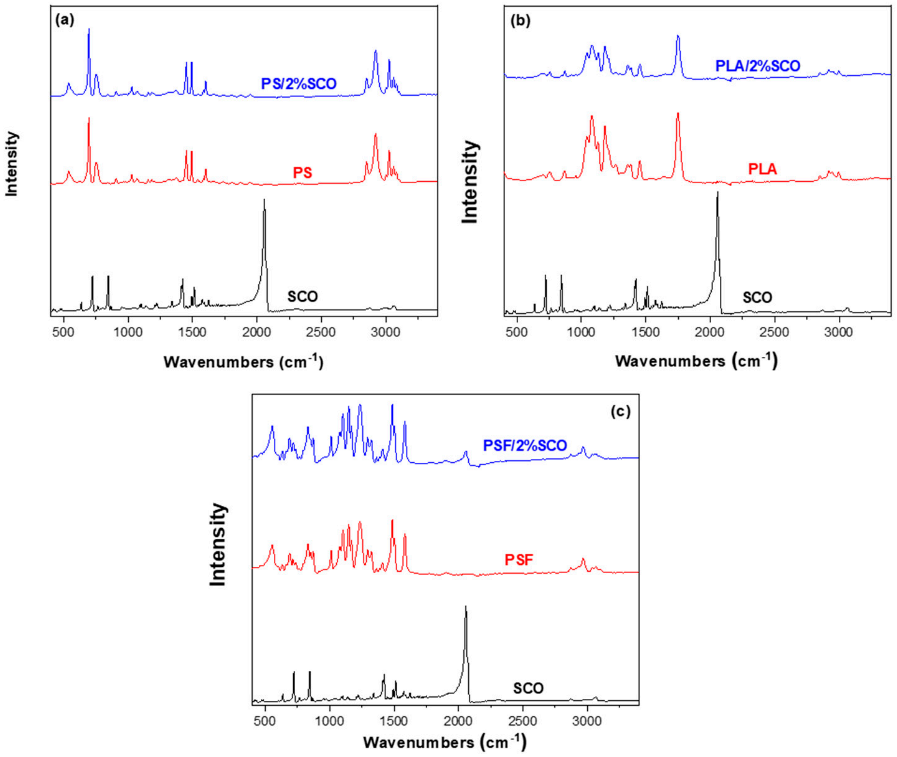

Figure S3. The ATR/FTIR spectra of the (a) PS, SCO and PS/2% SCO films, (b) PLA, SCO and PLA/2% SCO films, and (c) PSF, SCO and PSF/2% SCO films.

S3. Migration release study

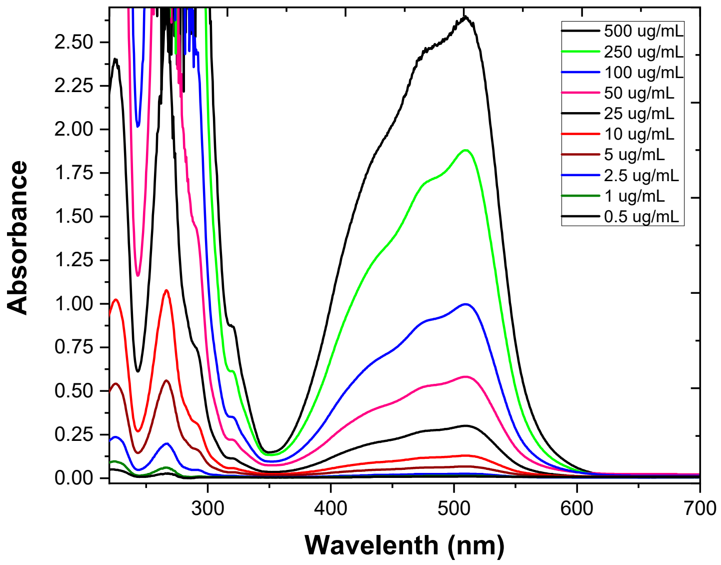

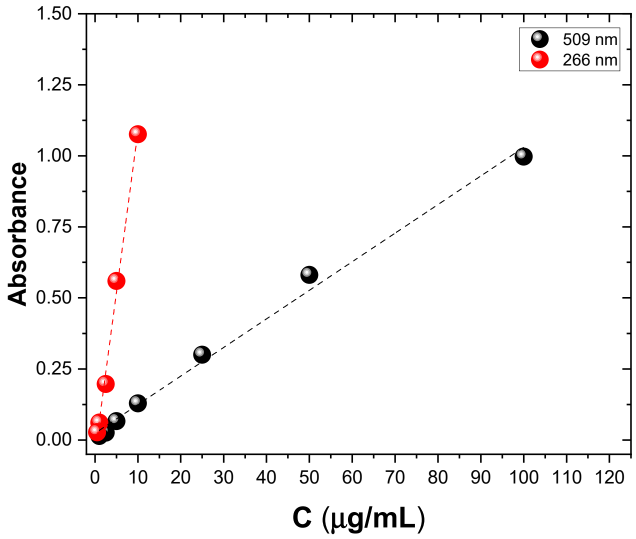

Figure S4. (a) UV/Vis spectra of the complex [Fe(1,10-phenanthroline)$_2$(NCS)$_2$] in 20 or 50% ethanol. The calculated maximum migration is at 100 μg/mL, which corresponds to an absorption of less than 1, (b) The calibration curve of [Fe(1,10-phenanthroline)$_2$(NCS)$_2$] in 20 or 50% ethanol.

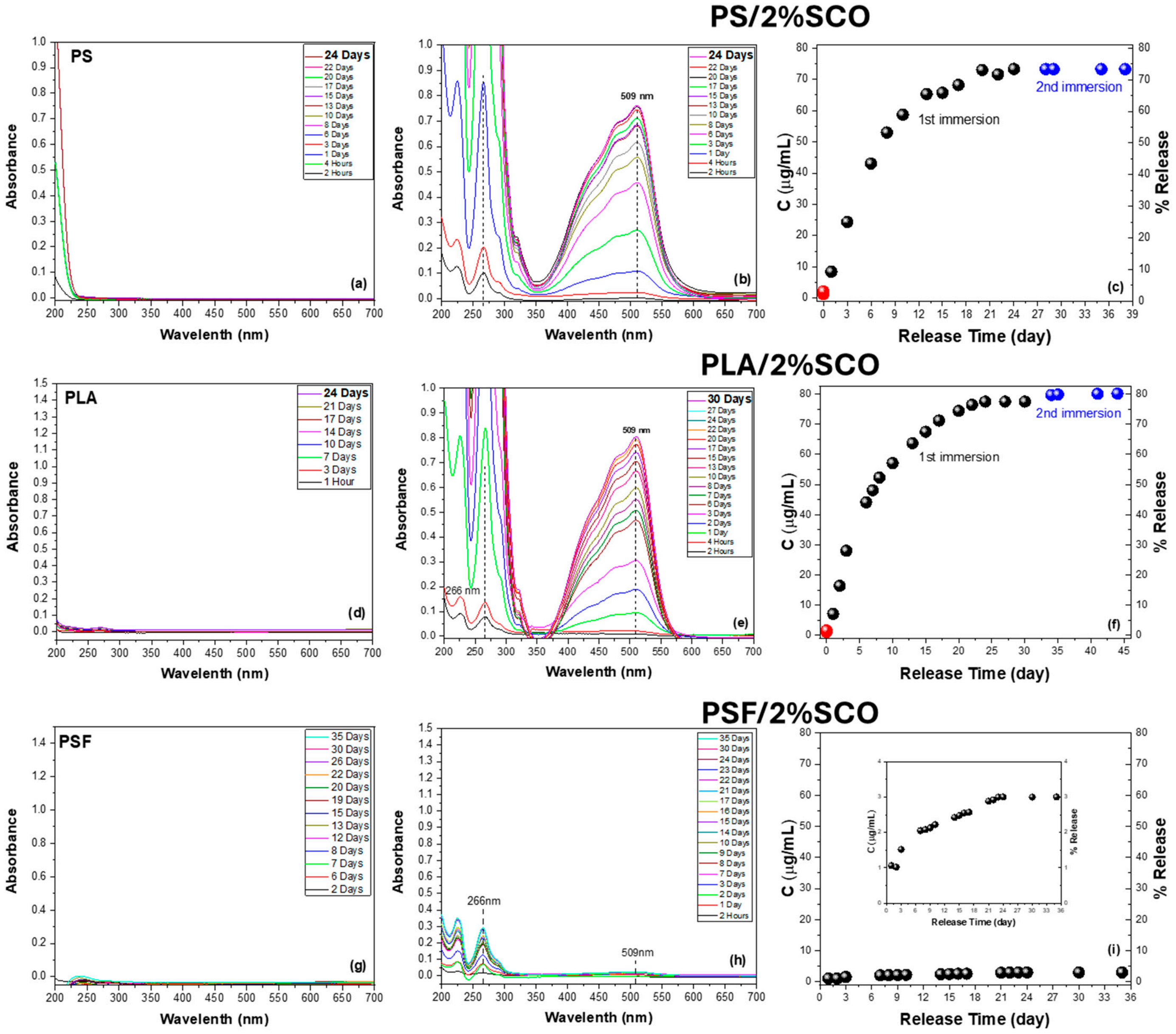

Figure S5. Migration release study of PS, PLA, PSF and the PS-SCO, PLA-SCO and PSF-SCO composites in 20% v/v ethanol. The UV/Vis spectra at different measurement times after immersion of (a) PS, (b) PS-SCO, (d) PLA, (e) PLA-SCO, (g) PSF, (h) PSF-SCO and the corresponding concentration (μg/mL) and percentage of the SCO release in the 20% v/v ethanol as a function of time (c, f, i).

S4.Post migration characterization

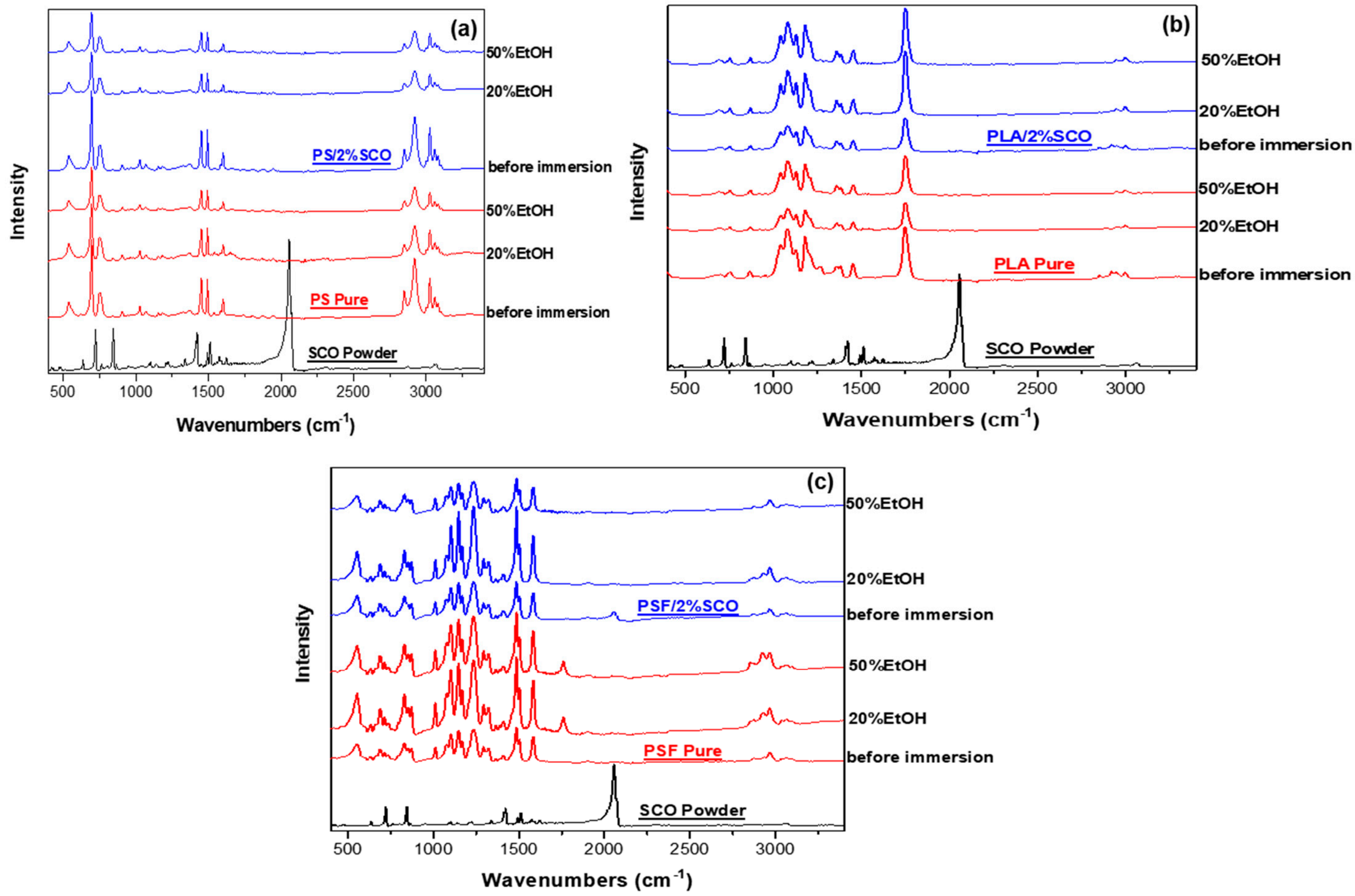

Figure S6. ATR/FTIR spectra before and after migration of PS and PS-SCO (a) PLA and PLA-SCO (b), PSF and PSF-SCO (c) composites.

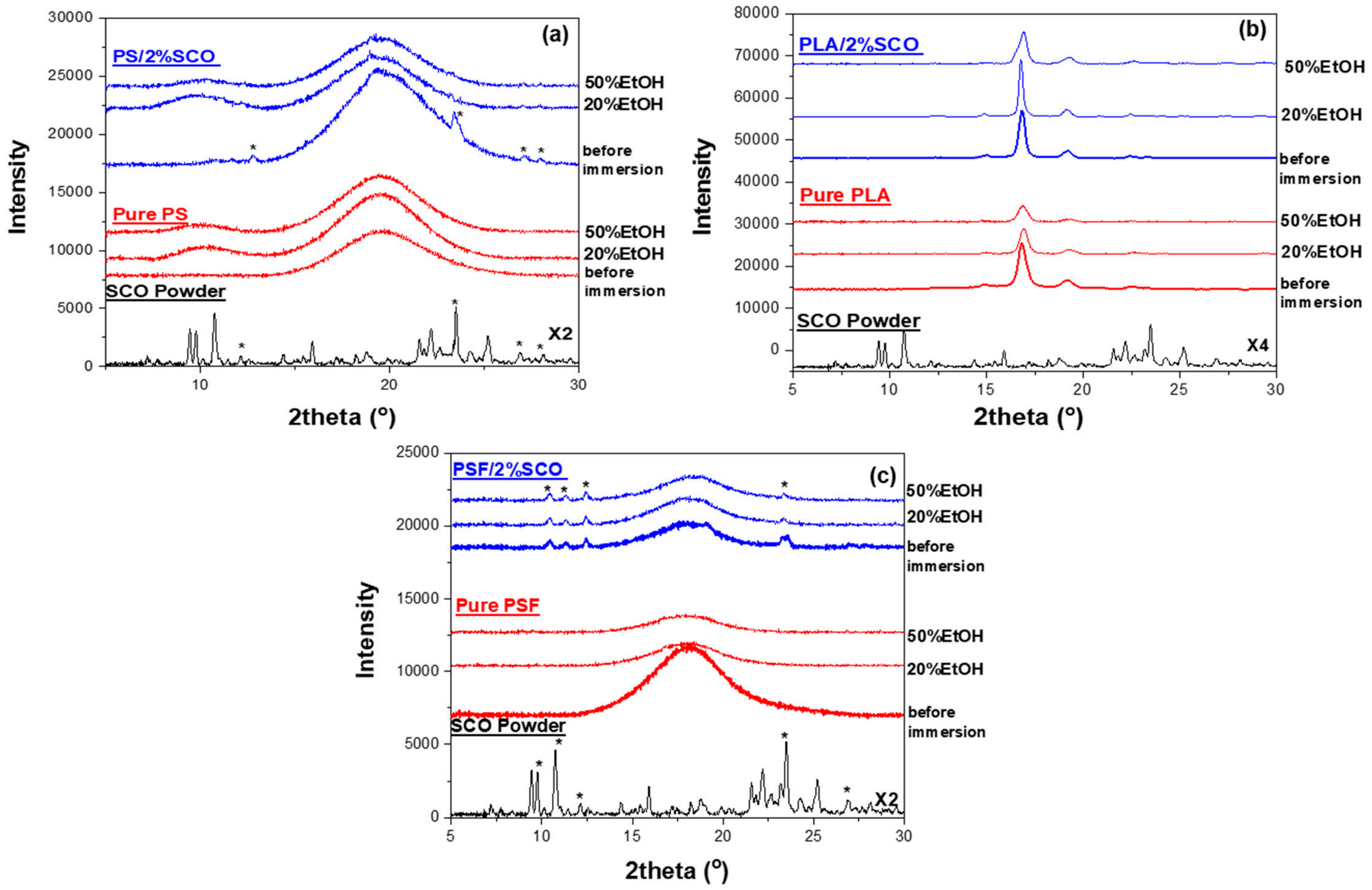

Figure S7. XRD before and after migration of PS and PS-SCO (a) PLA and PLA-SCO (b), PSF and PSF-SCO (c) composites.

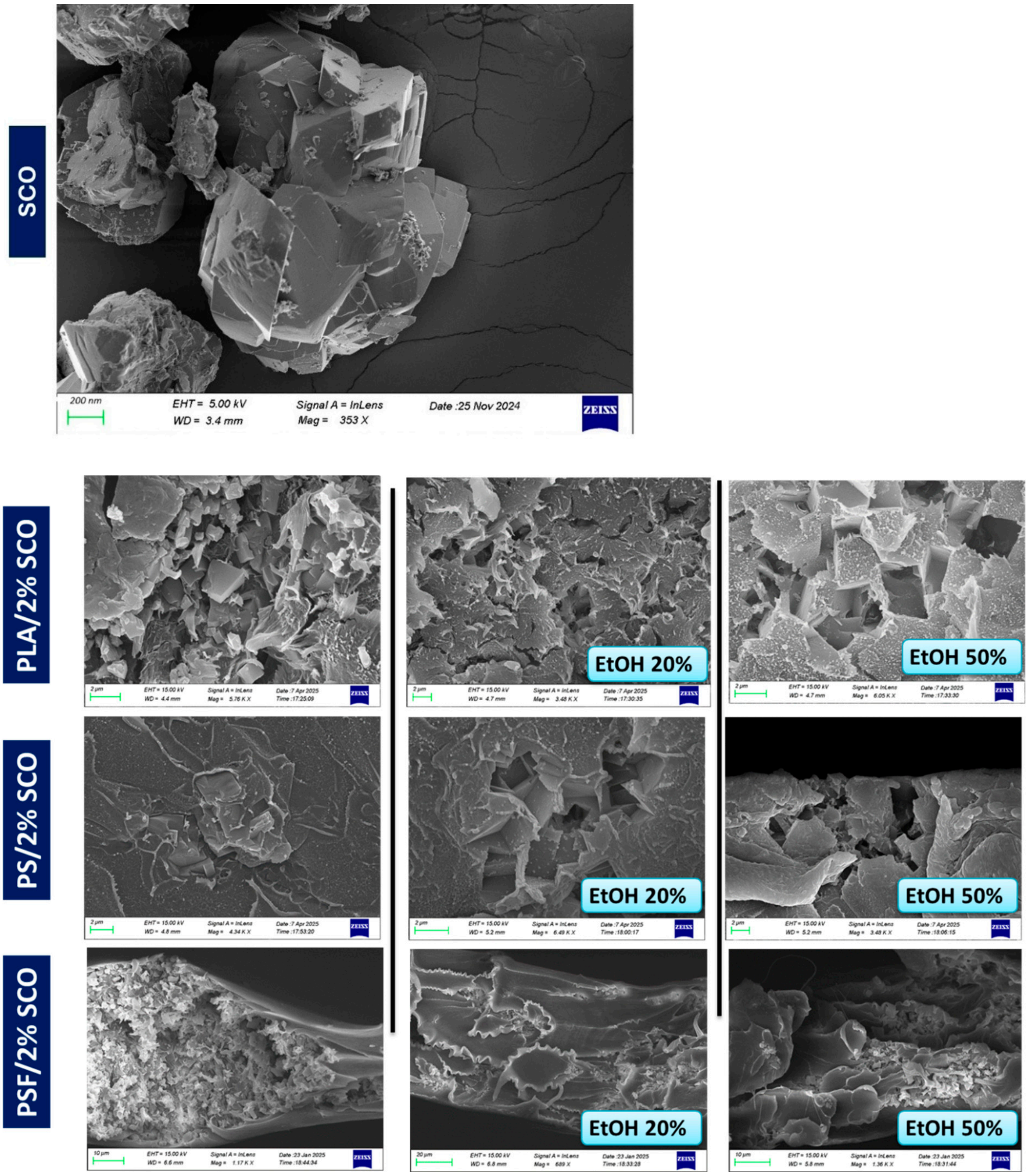

Figure S8. SEM images of the SCO particles of [Fe(1,10-phenanthroline)$_2$(NCS)$_2$], before and after migration of PS-SCO, PLA-SCO and PSF-SCO composites.

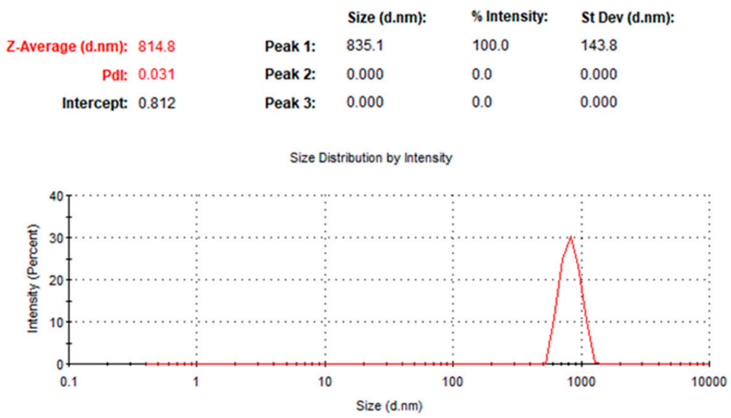

Figure S9. Calculated particle size distributions (with respect to intensity) obtained for the SCO particles of [Fe(1,10-phenanthroline)$_2$(NCS)$_2$] in CH_2Cl_2 resembling the film casting conditions. The extracted average hydrodynamic radius of SCO particles is denoted in the corresponding plot.
